# Decreasing case fatality rate following invasive pneumococcal disease, North East England, 2006–2016

**DOI:** 10.1017/S0950268819000657

**Published:** 2019-04-15

**Authors:** C. Houseman, K. E. Chapman, P. Manley, R. Gorton, D. Wilson, G. J. Hughes

**Affiliations:** 1 Field Service, Public Health England, Newcastle upon Tyne, UK; 2 Public Health England North East, Newcastle upon Tyne, UK; 3 Field Service, Public Health England, Leeds, UK

**Keywords:** Case fatality rate, invasive pneumococcal disease, mortality, pneumococcal infection, pneumococcal vaccine, *Streptococcus pneumoniae*

## Abstract

Declining mortality following invasive pneumococcal disease (IPD) has been observed concurrent with a reduced incidence due to effective pneumococcal conjugate vaccines. However, with IPD now increasing due to serotype replacement, we undertook a statistical analysis to estimate the trend in all-cause 30-day case fatality rate (CFR) in the North East of England (NEE) following IPD. Clinical, microbiological and demographic data were obtained for all laboratory-confirmed IPD cases (April 2006–March 2016) and the adjusted association between CFR and epidemiological year estimated using logistic regression. Of the 2510 episodes of IPD included in the analysis, 486 died within 30 days of IPD (CFR 19%). Increasing age, male sex, a diagnosis of septicaemia, being in ⩾1 clinical risk groups, alcohol abuse and individual serotypes were independently associated with increased CFR. A significant decline in CFR over time was observed following adjustment for these significant predictors (adjusted odds ratio 0.93, 95% confidence interval 0.89–0.98; *P* = 0.003). A small but significant decline in 30-day all-cause CFR following IPD has been observed in the NEE. Nonetheless, certain population groups remain at increased risk of dying following IPD. Despite the introduction of effective vaccines, further strategies to reduce the ongoing burden of mortality from IPD are needed.

## Introduction

Invasive pneumococcal disease (IPD), caused by infection with the bacterium *Streptococcus pneumoniae*, is one of the most common causes of bacteraemic pneumonia, septicaemia and meningitis worldwide, with a substantial global burden in young children, older adults and those with certain co-morbidities [1, 2]. The UK immunisation programme currently includes two pneumococcal vaccines [3]. The 13-valent pneumococcal conjugate vaccine (PCV13) was introduced in 2010 (2 + 1 schedule), directly replacing the seven-valent PCV introduced in 2006 for children. A single dose of 23-valent pneumococcal polysaccharide vaccine (PPV23) has been recommended for people at increased risk since 1992 and all individuals aged ⩾65 years since 2003. Immunisation coverage of PCV at 12 months has been consistently above 90% both nationally and across the North East of England (NEE) since 2009, whilst PPV coverage for persons ⩾65 years of age has consistently reached 70% (immunisation data available from www.gov.uk).

IPD incidence in NEE, and the UK as a whole, declined significantly after the introduction of PCV7 and PCV13 [4, 5]; reductions due to direct effects for those vaccinated and indirect (herd-level) effects for non-vaccinated age groups due to reduced carriage and transmission [6, 7]. However, the decline in PCV serotypes coincided with the emergence of non-PCV serotypes and the incidence of IPD increased for the first time in 2014/2015 and has done so consistently thereafter; suggesting the maximum benefit of the PCV programme may have been achieved [4, 5]. In addition to altered serotype dynamics, changes have been observed in the age profile of cases with increased incidence most pronounced in older age groups [4, 5].

Evidence suggests that case fatality rates (CFR) following IPD have declined following the introduction of PCVs [8–14]. However, published studies have either included only specific age groups [9, 12] or have not fully adjusted for individual-level risk factors and/or undertaken a full trend analysis [8, 10, 11, 13, 14]. Using enhanced surveillance data from a region of England, we estimated the trend in 30-day all-cause CFR following IPD over a 10-year period after fully adjusting for known individual risk factors.

## Methods

### Study population

The NEE has a relatively stable population of about 2.6 million persons, which includes approximately 147 000 children aged <5 years and 457 000 adults aged over 64 years (population data available from: www.ons.gov.uk). The NEE covers an area of approximately 8600 km^2^ and comprises 12 local authorities.

## Surveillance data and linkage to death data

Surveillance data for cases of IPD with a specimen date between 1 April 2006 and 31 March 2016 were obtained from the North East IPD Enhanced Surveillance System (IPDESS). This system has been in operation since April 2006; full details of the surveillance system are described elsewhere [15]. In brief, all laboratory-confirmed cases of IPD are notified to the local health protection team and telephone interviews with laboratory and clinical staff are conducted to obtain details of risk factors and pneumococcal immunisation history. Typing is undertaken at the national reference laboratory. Cases are residents of NEE with *S. pneumoniae* detected from a normally-sterile site (e.g. blood, cerebrospinal fluid) and clinical diagnosis of IPD. Cases were linked to the Office for National Statistics registered deaths up to 30 December 2016 using unique National Health Service numbers.

### Statistical analysis

A binary logistic regression model was used to estimate adjusted independent associations between epidemiological year of diagnosis (April–March, retained in the model throughout) and CFR (30-day all-cause) following IPD. A forward selection model-building approach was used, starting from the base model including epidemiological year. Additional variables were added into the model one at a time in order of decreasing statistical significance from single-variable analysis and the added covariate retained if the *P* value of the added variable and improvement in model fit using a likelihood ratio test (LRT) were both <0.05. Covariates considered were: age, sex, clinical diagnosis (bacteraemic pneumonia, septicaemia, meningitis, other), clinical risk factor (one or more of the following: chronic heart disease, chronic lung disease, chronic liver disease, chronic renal disease, diabetes, immunosuppression, asplenia, cochlear implant, cerebrospinal fluid leak), alcohol misuse, immunisation status, vaccine-type serotype subgroup (PCV7: 4, 6B, 9V, 14, 18C, 19F, 23F; PCV13-exclusive: 1, 3, 5, 6A, 7F, 19A; PPV23-exclusive: 2, 8, 9N, 10A, 11A, 12F, 15B, 17F, 20, 22F, 33F; non-vaccine type, NVT: all other serotypes) and individual pneumococcal serotypes which each accounted for ⩾1% of cases (included as a single categorical variable). Where no risk factor data were available, cases were coded as having no clinical risk factors or alcohol abuse.

Goodness of fit of the final model (where all parameters had an associated *P* value <0.05) was assessed using the Hosmer–Lemeshow goodness of fit test (10 groups) and Receiver Operating Characteristic curve [16]. A multivariable fractional polynomial model of the final logistic regression model was used to test the assumption of linearity for epidemiological year and age [17]. Adjusted CFR was estimated using predictions from the final model with mean values for other covariates.

### Statistical software

All analyses were performed using Stata v13.1 (StataCorp., College Station, Texas, USA).

## Results

### Surveillance data and linkage to death data

After exclusion of four cases who were either not permanent residents of NEE (*n* = 2) or where data within IPDESS were inconsistent with that retrieved during linkage to death data (*n* = 2), a total of 2510 IPD cases were included in the analysis (Table 1).

**Table 1.** Characteristics of invasive pneumococcal disease cases and 30-day all-cause case fatality rate in North East England, April 2006–March 2016

Characteristic	Category	*N* (%)	Deaths (CFR)	*P*^a^
All cases	–	2510	486 (19)	
Age group	<5 years	230 (9)	8 (3)	<0.001
	5–64 years	1128 (45)	132 (12)	
	>64 years	1152 (46)	346 (30)	
Sex	Female	1234 (49)	221 (18)	0.077
	Male	1276 (51)	265 (21)	
Clinical diagnosis	Pneumonia^b^	1676 (67)	300 (18)	<0.001
	Septicaemia	276 (11)	83 (30)	
	Meningitis	260 (10)	39 (15)	
	Other/not specified	298 (12)	64 (21)	
Risk factors^c^	No risk factors	1166 (46)	151 (13)	<0.001
	⩾1 risk factor	1344 (54)	335 (25)	
Alcohol misuse	No	2292 (91)	431 (19)	0.025
	Yes	218 (9)	55 (25)	
Vaccination history	No	1252 (50)	177 (14)	<0.001
	Yes	1132 (45)	271 (23)	
	Not known	126 (5)	38 (30)	
Vaccine-type serotype subgroup	PCV7	262 (10)	60 (23)	0.098
	PCV13-exclusive	831 (33)	152 (18)	
	PPV23-exclusive	750 (30)	137 (18)	
	NVT	364 (15)	85 (23)	
	Not serotyped	303 (12)	52 (17)	
Serotype	1	198 (9)	12 (6)	<0.001
	3	189 (9)	56 (30)	
	4	42 (2)	7 (17)	
	6A	40 (2)	14 (35)	
	6B	30 (1)	9 (30)	
	6C	58 (3)	16 (28)	
	7F	222 (10)	30 (14)	
	8	231 (10)	25 (11)	
	9N	57 (3)	15 (26)	
	9V	37 (2)	10 (27)	
	10A	34 (2)	6 (18)	
	11A	47 (2)	18 (38)	
	12F	96 (4)	10 (10)	
	14	45 (2)	8 (18)	
	15A	46 (2)	11 (24)	
	16F	26 (1)	8 (31)	
	18C	27 (1)	3 (11)	
	19A	176 (8)	39 (22)	
	19F	39 (2)	14 (36)	
	20	36 (2)	8 (22)	
	22F	154 (7)	32 (21)	
	23A	46 (2)	12 (26)	
	23F	42 (2)	9 (21)	
	31	38 (2)	15 (39)	
	33F	55 (2)	12 (22)	
	35F	31 (1)	6 (19)	
	Other	165 (7)	29 (18)	

a Fisher's exact test.

b Bacteraemic pneumonia.

c Chronic heart disease, chronic lung disease, chronic liver disease, chronic renal disease, diabetes, immunosuppression, asplenia, cochlear implant, cerebrospinal fluid leak.

CFR, case fatality rate (%); PCV7, seven-valent pneumococcal conjugate vaccine; PCV13, 13-valent pneumococcal conjugate vaccine; PPV23, 23-valent pneumococcal polysaccharide vaccine.

Of the 2510 cases included in the multivariable analysis, corresponding to an average annual incidence rate of 9.7 (range 6.5–12.1) per 100 000 population, 486 cases had a date of death within 30 days of their specimen date (average annual CFR 19%, range 16–24%). Serotype was available for 88% of cases; PCV13 serotypes accounting for 50%, PPV23-exclusive serotypes 34% and NVT serotypes 16%. Twenty-six serotypes each accounted for >1% of cases (⩾26 cases per serotype) (Table 1).

### Multivariable analysis

The final multivariable model (2192 cases; 303 cases excluded with unknown serotype and 15 cases excluded as main diagnosis not recorded) for association with 30-day all-cause mortality contained epidemiological year, age, sex, clinical diagnosis, presence of clinical risk factors, alcohol misuse and pneumococcal serotype (Table 2). This model had an overall acceptable goodness of fit (Hosmer–Lemeshow *χ*^2^ 7.89, degrees of freedom (DF) 8, *P* = 0.444), improved fit compared to the starting model (LRT *χ*^2^ = 330.52, DF 34, *P* < 0.001) and acceptable discrimination of cases (area under the Receiver Operating Characteristic curve 0.77).

**Table 2.** Associations between characteristics of invasive pneumococcal disease cases and 30-day all-cause fatality rate in North East England, April 2006–March 2016

Characteristic	Category	aOR (95% CI)	*P*	Overall *P*^a^
Epidemiological year^b^	–	0.93 (0.89–0.98)	0.003	–
Age in years	–	1.04 (1.03–1.05)	<0.001	–
Sex	Female	Ref.	–	–
	Male	1.57 (1.24–1.98)	<0.001	
Clinical diagnosis	Pneumonia^c^	Ref.	–	<0.001
	Septicaemia	2.30 (1.64–3.21)	<0.001	
	Meningitis	1.43 (0.89–2.28)	0.135	
	Other	1.29 (0.88–1.89)	0.197	
Clinical risk factors^d^	No risk factors	Ref.	–	–
	⩾1 risk factor	1.36 (1.05–1.75)	0.020	
Alcohol misuse	No	Ref.	–	–
	Yes	2.48 (1.67–3.68)	<0.001	
Pneumococcal serotype	Other	Ref.	–	0.006
	1	0.60 (0.28–1.29)	0.192	
	3	1.97 (1.14–3.40)	0.014	
	4	1.02 (0.39–2.69)	0.968	
	6A	2.39 (1.04–5.52)	0.041	
	6B	1.40 (0.52–3.71)	0.504	
	6C	1.55 (0.74–3.25)	0.245	
	7F	1.10 (0.60–1.99)	0.766	
	8	0.76 (0.41–1.40)	0.376	
	9N	1.87 (0.88–4.01)	0.106	
	9V	1.32 (0.54–3.23)	0.540	
	10A	1.12 (0.40–3.13)	0.828	
	11A	2.41 (1.13–5.14)	0.022	
	12F	0.72 (0.32–1.60)	0.418	
	14	0.74 (0.29–1.89)	0.535	
	15A	1.34 (0.59–3.07)	0.485	
	16F	1.76 (0.65–4.79)	0.267	
	18C	0.68 (0.18–2.66)	0.584	
	19A	1.52 (0.85–2.70)	0.155	
	19F	3.30 (1.41–7.74)	0.006	
	20	1.21 (0.47–3.11)	0.694	
	22F	1.01 (0.55–1.86)	0.968	
	23A	1.33 (0.59–3.01)	0.488	
	23F	0.94 (0.37–2.40)	0.904	
	31	2.56 (1.13–5.76)	0.023	
	33F	1.46 (0.65–3.28)	0.365	
	35F	0.96 (0.34–2.69)	0.943	

a Wald test.

b April–March.

c Bacteraemic pneumonia.

d Chronic heart disease, chronic lung disease, chronic liver disease, chronic renal disease, diabetes, immunosuppression, asplenia, cochlear implant, cerebrospinal fluid leak.

aOR, adjusted odds ratio; Ref, reference group.

**Fig. 1.**
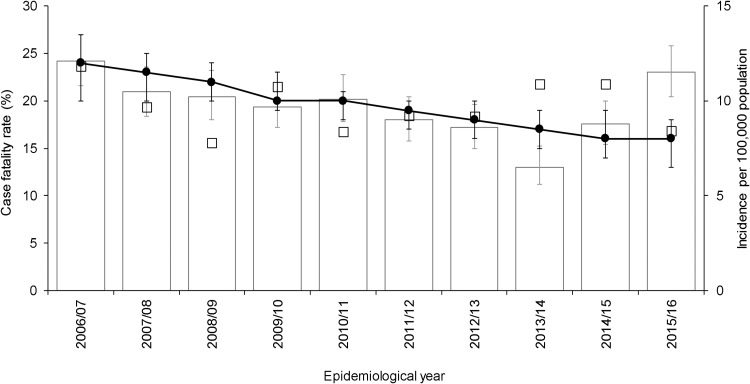
Crude 30-day all-cause case fatality rate, adjusted average predicted probabilities and incidence of invasive pneumococcal disease by epidemiological year (April–March) in North East England, April 2006–March 2016. Bars show incidence. Line indicates average predicted probability of 30-day all-cause mortality for cases presented as case fatality rate (CFR), adjusted for age, sex, clinical presentation, risk factors and pneumococcal serotype. Epidemiological year is April–March. Open squares indicate crude CFR. Error bars indicate 95% confidence intervals.

After adjustment for other independent predictors, 30-day all-cause mortality demonstrated a small but significant decrease by epidemiological year (adjusted odds ratio (aOR) 0.93, 95% confidence interval (CI) 0.89–0.98, *P* = 0.003), corresponding to a decline in adjusted average CFR from 24% (20–27%) in the epidemiological year 2006/2007 to 16% (13–18%) in the epidemiological year 2015/2016 (Fig. 1). Although a multivariable fractional polynomial model indicated significant improvement of fit of the final model with a non-linear transformation of age (*P* = 0.014), this did not result in substantial changes to the aOR of covariates (median change 2.0%, range 0.27–14.39%) and all covariates retained statistical significance. Importantly, the aOR for epidemiological year differed by just 0.52% (aOR 0.93, 95% CI 0.89–0.98).

A multivariable fractional polynomial model indicated no significant non-linearity in the association between 30-day all-cause mortality and epidemiological year (*P* > 0.05).

**Fig. 2.**
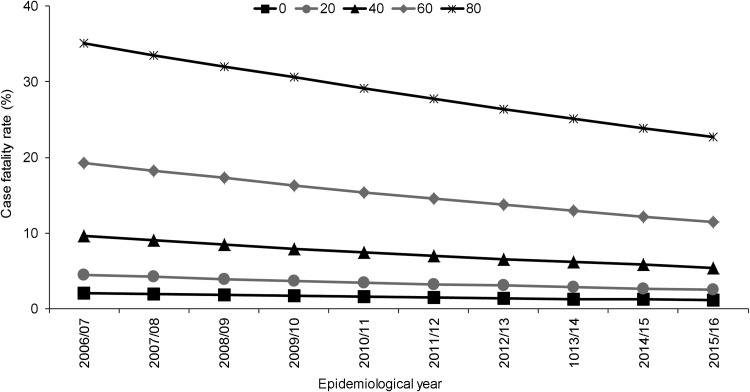
Adjusted predicted probabilities of 30-day all-cause case fatality rate by epidemiological year (April–March) in North East England by age, April 2006–March 2016. Lines indicate predicted probability of 30-day all-cause mortality presented as case fatality rate (CFR) for individuals of specific ages (years) adjusted for sex, clinical presentation, risk factors and pneumococcal serotype using mean values for covariates. Epidemiological year is April–March.

Independent to the association with epidemiological year, increasing age, male sex, septicaemia, presence of ⩾1 clinical risk factor, alcohol misuse and pneumococcal serotype were significant predictors of increased 30-day all-cause mortality. Of the individual serotypes included, serotype 1 had the lowest crude CFR (6%) and serotype 31 the highest (39%) (Table 1). Serotype 1 also had the lowest aOR (0.60) although this was not statistically significant (Table 2). Although no significant interaction was observed between the annual trend in 30-day all-cause mortality and age groups (<5, 5–64, ⩾65 years, *P* = 1.000) or age quintiles (*P* = 0.103), the pattern was expectedly more pronounced for older ages (Fig. 2).

## Discussion

Despite recent increases in IPD in NEE in the epidemiological years 2014/2015 and 2015/2016, following a sustained period of reducing incidence [4], our study provides reassuring evidence that this increased incidence does not seem to be associated with the emergence of serotypes associated with worse outcomes. Moreover, a decline in adjusted CFR has continued. Our study provides evidence of decreasing CFR following IPD and, to the best of our knowledge, has for the first time estimated the long-term trend in CFR following IPD after adjustment for known individual risk factors.

Given that the rise in IPD incidence in NEE has not disrupted a general decreasing trend in adjusted CFR, it is not surprising that only one (9N) of the six emergent serotypes seen in our population (8, 9N, 12F, 15A, 23A, 25F) [4] has been previously associated with increased CFR in NEE [18]. Of the four serotypes previously shown to be significantly associated with increased CFR following IPD in NEE [18], 9N is the only serotype where the results of this current study are not consistent with those of the earlier study and the reason for this requires further exploration. The observation of rising incidence with declining CFR is consistent with the hypothesised relationship between increasing invasiveness and reduced virulence – those serotypes that have emerged as causative agents of IPD in NEE in recent years represent primary pathogens with an intrinsically low CFR compared to other more heavily encapsulated serotypes which function as opportunistic pathogens causing severe disease in those with underlying risk factors [19]. Certainly, the increasing prevalence of the low CFR serotype 8 [18–20] in NEE [4] further supports this hypothesis.

The reasons for the existence of a significant relationship between epidemiological year and declining CFR, after adjusting for risk factors and infecting serotype (all of which have been observed and described elsewhere [18, 20, 21, 22]), requires further exploration. Factors may include: improved diagnostic and laboratory practices (that may have led to disproportionate identification of uncomplicated cases [11], or earlier diagnoses with better prognosis); improved treatment for risk factors associated with CFR (thus reducing their relative impact on CFR following IPD); reduced prevalence of smoking [23]; and/or improved treatment of IPD. Certainly there is evidence for a declining prevalence of smoking in NEE (https://fingertips.phe.org.uk/profile/tobacco-control) but we were unable to adjust for smoking at an individual level due to limited data availability.

Our study indicates that a small but significant trend in declining 30-day all-cause CFR following IPD has been observed in NEE between 2006 and 2016. This suggests that selection pressure due to the use of effective vaccines has not led to the emergence of serotypes of *S. pneumoniae* that are associated with more severe outcomes. The existence of a declining CFR raises interesting questions related to trends in case ascertainment bias that require further investigation. Despite this encouraging finding, particularly following recent increases in IPD incidence, certain risk groups have an increased probability of dying following IPD and work should continue to reduce the burden of mortality and morbidity from IPD.
